# An Index of Diseases and Their Treatment

**Published:** 1877-12

**Authors:** 


*An Index of Diseases and their Treatment.* By Thomas Hawkes Tanner, M. D., F. L. S. Second Edition. Revised by W. H. Broadbent, M. D. Philadelphia: Lindsay & Blakiston. 1877. Buffalo: T. H. Butler. pp. 432. Price, $3.00.

We notice these two books together not in order to compare them but rather from the fact that they are in a measure complements of each other. Although covering in part the same ground the view is so different that there is no redundance. The possession of one does not render the other useless, but rather one will enhance the value of the other. This will be clearly seen by a cursory view of their contents.

The physician is often at a loss to know where to look for desired information in the treatment of disease. It will be found scattered through elaborate and voluminous text books and treatises, or in the current periodical medical literature but to the busy practitioner these are often a sealed page since they are in many instances inaccessible. It is to supply this demand that Drs. Dunglison and Tanner have labored. The first has the following heads. General information as to weights, measures, themometers, and the solubility of medicines; therapeutic and practical hints, doses of medicines, incompatibles, *the modern treatment of diseases with the remedies applicable, selected prescriptions.* Examination of urine, poisons and disinfectants, etc., dietetic rules, composition of various articles of food, dietetic preparations, and special forms of diet; how to conduct a post-mortem examination.

The scope of the second work may be seen from the following quotation: "In constructing the various articles of which this index is composed, the author has endeavored by giving a brief description of each disease to make its diagnosis sure. With regard to the sections of treatment, it is to be remembered that the numbers appended to the drugs not only refer to the formulæ, but indicate those remedies on which it is believed that reliance should be chiefly placed. As a rule, however, most of the agents which have been recommended by different authors are mentioned; although where they are not deemed particularly useful, either no reference is given for the mode in which they are to be prescribed, or they are placed in a separate paragraph." It is divided into two portions, the body of the work giving a brief description and mode of treatment of each disease as taken up in alphabetical order and an appendix containing nearly five hundred formulæ. It covers exactly the same ground that the portion of the first work which we have indicated in title by italics. But in the first no description of a disease is given, nor is there any indication of the special adaptedness or mode of administering suggested. The whole topic being contained upon forty-one pages while in the index two hundred and eighty pages are devoted to their delineation.

To many physicians the diagnosis is fully as essential as the remedy to be administered but the value and relative efficacy of a certain drug in a particular disease may even rank higher in his esteem than either. These are both found in the Index but not in the Reference book. Thus have we shown that the Index is the complement of the Reference Book filling out in detail what is presented in skeleton in the latter.

The Reference Book contains by far the most important and practical information but the Index will prove equally useful to the active practitioner, perhaps it will be found the greater assistant. The Reference book may be made of great use in following out the treatment of any disease. Thus having but a few minutes to look up the treatment of a case which he has had for some time but which does not improve under treatment, the physician will take up the Index and by a glance make sure of his diagnosis, then he looks at the treatment, and finds one or two remedies which he has not as yet tried. He now refers to the Reference Book and from the tables of solubilities, incompatibles and doses, easily determines the manner in which the remedies are best administered. The Reference Book now assists the Index.

We have presented in brief some of the many merits of these two valuable works both of which are indespensible companions of the young and inexperienced, but active members of the profession. We can recommend them to every physician; no Doctor's library would be complete without them. Few volumes contain so much that is of practical importance as do these two small works: we know of none that would be of more use to the man beginning practice. C.

